# Supplementary material for: A Descriptive Study of 103 Primary Cutaneous B-Cell Lymphomas: Clinical and Pathological Characteristics and Treatment from the Spanish Lymphoma Oncology Group (GOTEL)
Source: Cancers (Basel). 2024 Mar 3;16(5):1034. doi: 10.3390/cancers16051034 (PMC10931196; doi:10.3390/cancers16051034)

Supplementary Information Cancers 2893059

Table S1: Percentiles for age

**Characteristic**	**Overall**, N = 103[1]	**PCMZL**, N = 68[1]	**PCFCL**, N = 27[1]	**PCDLBCL-LT**, N = 8[1]
Age	53 (40, 65)	51 (38, 60)	56 (50, 66)	66 (58, 74)

[1]Median (IQR)

Table S2: HANS algorithm for PCDLBCL-LT

**Characteristic**	**DLBCL leg type**, N = 8[1]
Immunophenotype	
GCB	3 (38%)
non-GCB	4 (50%)
unknown	1 (13%)

[1]n (%)

Table S3: Available immunophenotypes (all group)

**Characteristic**	**Overall**, N = 103[1]	**PCMZL**, N = 68[1]	**PCFCL**, N = 27[1]	**PCDLBCL-LT**, N = 8[1]
Immunophenotype				
CD10- CD3- CD5- CiclinaD1- CD30- ALK- CD20+ CD79a+ BCL2+ BCL6+ (75%) MUM1+ (50%), IgM, Ki67 (90%)	1 (1.0%)			1 (13%)
CD20- CD10- BCL6+ BCL2+ MVMI- C79a+	1 (1.0%)		1 (3.7%)	
CD20+ BCL2- BCL6+ CD3-	1 (1.0%)		1 (3.7%)	
CD20+ BCL2- BCL6+ CD79a+	1 (1.0%)		1 (3.7%)	
CD20+ BCL2+ BCL6+ MUM1+	1 (1.0%)			1 (13%)
CD20+ BCL2+ CD79a+	1 (1.0%)		1 (3.7%)	
CD20+ BCL6+	1 (1.0%)		1 (3.7%)	
CD20+ CD10+ bcl2- bcl6+ CD23+	1 (1.0%)		1 (3.7%)	
CD20+ CD10+ Bcl2- Bcl6+ CD3+	1 (1.0%)		1 (3.7%)	
CD20+ CD10+ BCL2- BCL6+ CD5- CD43- CD1-	1 (1.0%)		1 (3.7%)	
CD20+ CD10+ BCL2+ BCL6+	1 (1.0%)		1 (3.7%)	
CD20+ CD10+ BCL2+ BCL6+ CD5+ CD43+ CICLINA D1+	1 (1.0%)		1 (3.7%)	
CD20+ CD10+ BCL2+ BCL6+ CD79+	1 (1.0%)		1 (3.7%)	
CD20+ CD10+ BCL2+ BCL6+ KI67 35%	1 (1.0%)		1 (3.7%)	
CD20+ CD10+ BCL2+ CD3- Ki67 70%	1 (1.0%)		1 (3.7%)	
CD20+ CD79A+ CD10+ BCL2+ BCL6+ CD3- CD5- CD30- CD23- CD43-	1 (1.0%)			1 (13%)
CD20+ IgG LAMBDA P53+ CD5- CD3- CD43- CD30- CD10- BCL2- BCL6+ MUM1-	1 (1.0%)			1 (13%)
CD20+, BCL2+, MUM1+, CD30+	1 (1.0%)			1 (13%)
CD20+, CD3+	1 (1.0%)	1 (1.5%)		
CD20+, CD5+, CD43+, CD23 FOCAL, BCL2+	1 (1.0%)	1 (1.5%)		
CD20+, CD79+, PAX5+, Bcl2+ y Bcl6+; Ki67: 70-80%; CD10 y MUM1: positivo focal	1 (1.0%)			1 (13%)
CD20+, CD79A+, PAX5+, BCL6+, BCL2-, CD30-, CD10-, MUM1+	1 (1.0%)			1 (13%)
CD3+, CD5+, CD20+, CD79+, CD30+	1 (1.0%)	1 (1.5%)		
Not available or unknown	80 (78%)	65 (96%)	14 (52%)	1 (13%)

[1]n (%)

Table S4: Known immunophenotype by year of diagnosis.

Characteristic	PCMZL		PCFCL		PCDLBCL-LT	
	≤ 2005 N = 23[1]	> 2005 N = 45[1]	≤ 2005 N = 9[1]	> 2005 N = 18[1]	≤ 2005 N = 2[1]	> 2005 N = 6[1]
Immunophenotype						
Known or partial immunophenotype	2 (9%)	1 (2%)	1 (11%)	12 (67%)	2 (100%)	5 (83%)
Not available or unknown	21 (91%)	44 (98%)	8 (89%)	6 (33%)	0 (0%)	1 (17%)

[1]n (%)

Table S5: Results for Borrelia

Characteristic	**Overall**, N = 103[1]	**PCMZL**, N = 68[1]	**PCFCL**, N = 27[1]	**PCDLBCL-LT**, N = 8[1]
Borrelia Burgdorferi				
Positive	1 (1.0%)	1 (1.5%)	0 (0%)	0 (0%)
Negative	17 (17%)	15 (22%)	2 (7.4%)	0 (0%)
Unknown	85 (83%)	52 (76%)	25 (93%)	8 (100%)

[1]n (%)

Figure S1: Kaplan–Meier survival curves according to tumour histology (columnwise) and clinical response. **A**, progression-free survival; **B**, disease-specific survival; **C**, overall survival.

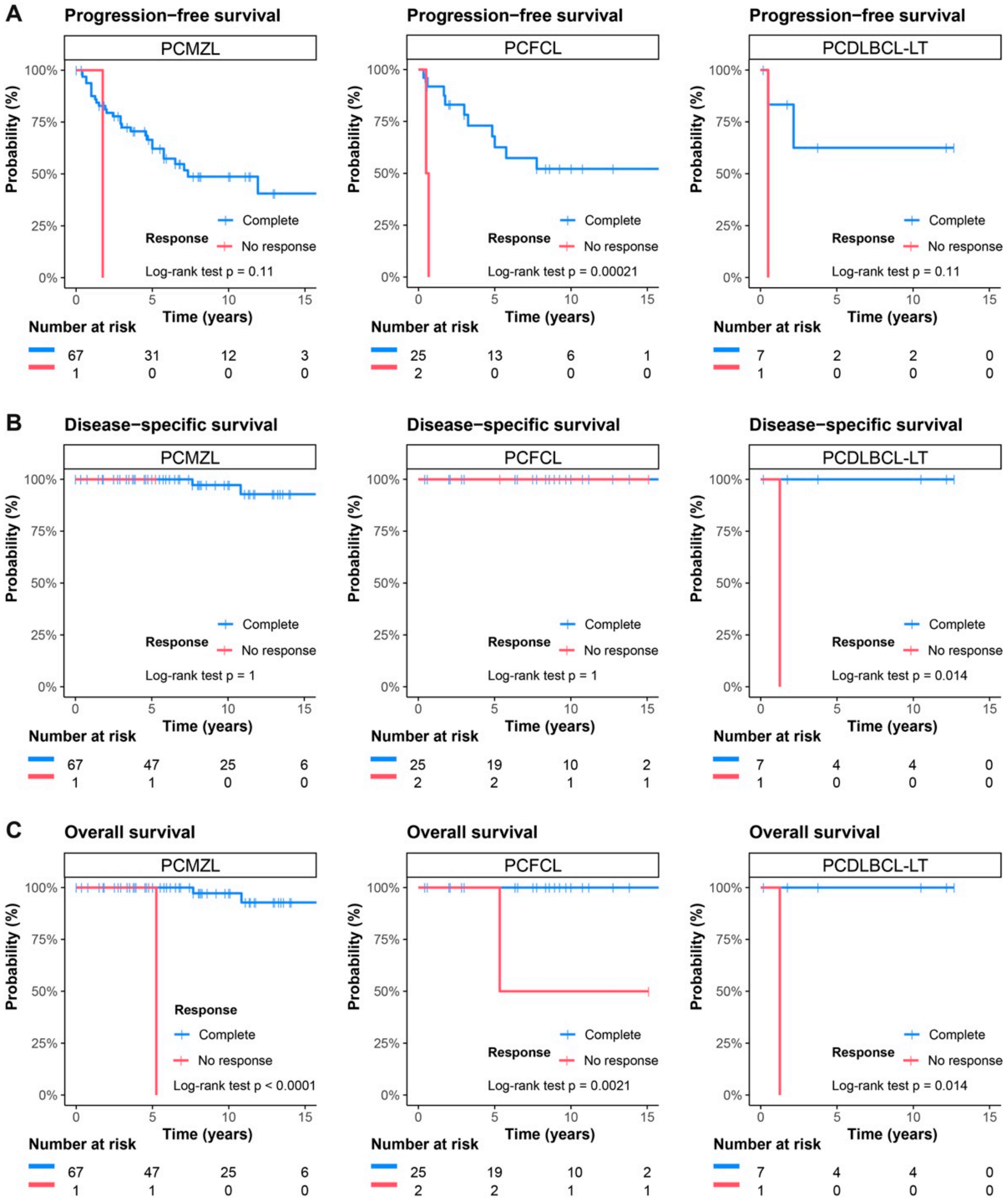

Figure S2: K–M survival curves for patients who received local treatment (strata) and by tumour histology (columnwise). **A**, progression-free survival; **B**, disease-specific survival; **C**, overall survival.

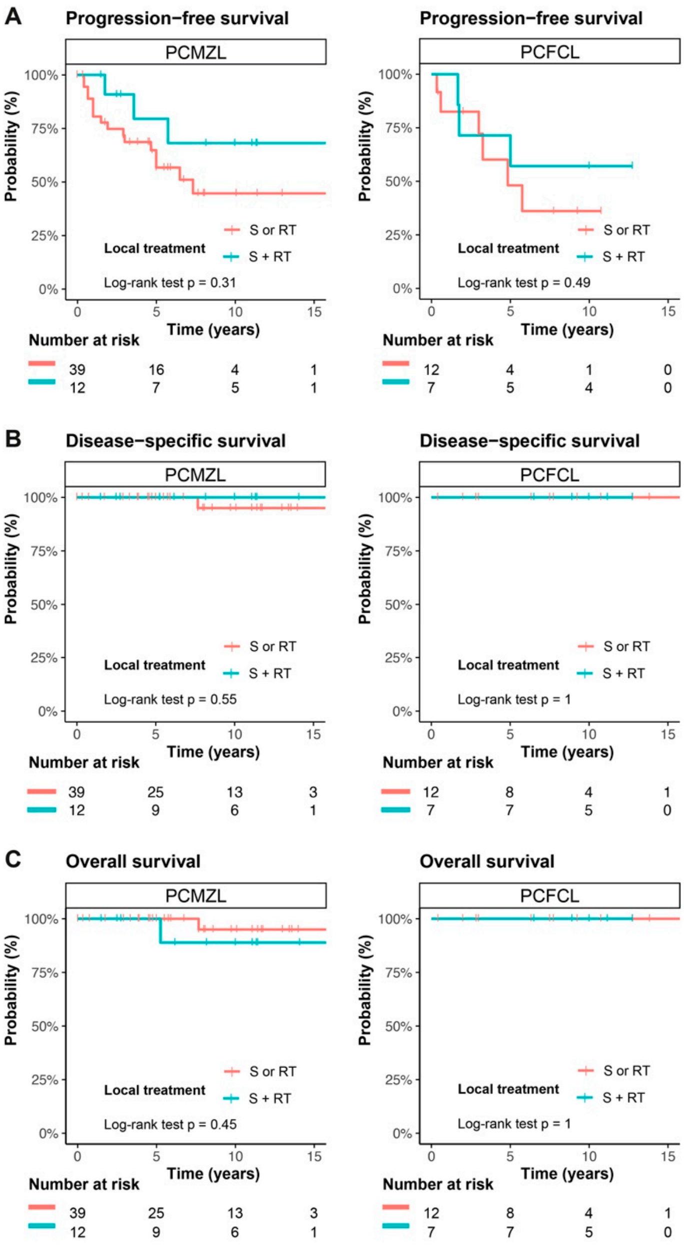

Figure S3: Kaplan–Meier survival curves according to tumour histology (columnwise) and tumour extension. **A**, progression-free survival; **B**, disease-specific survival; **C**, overall survival.

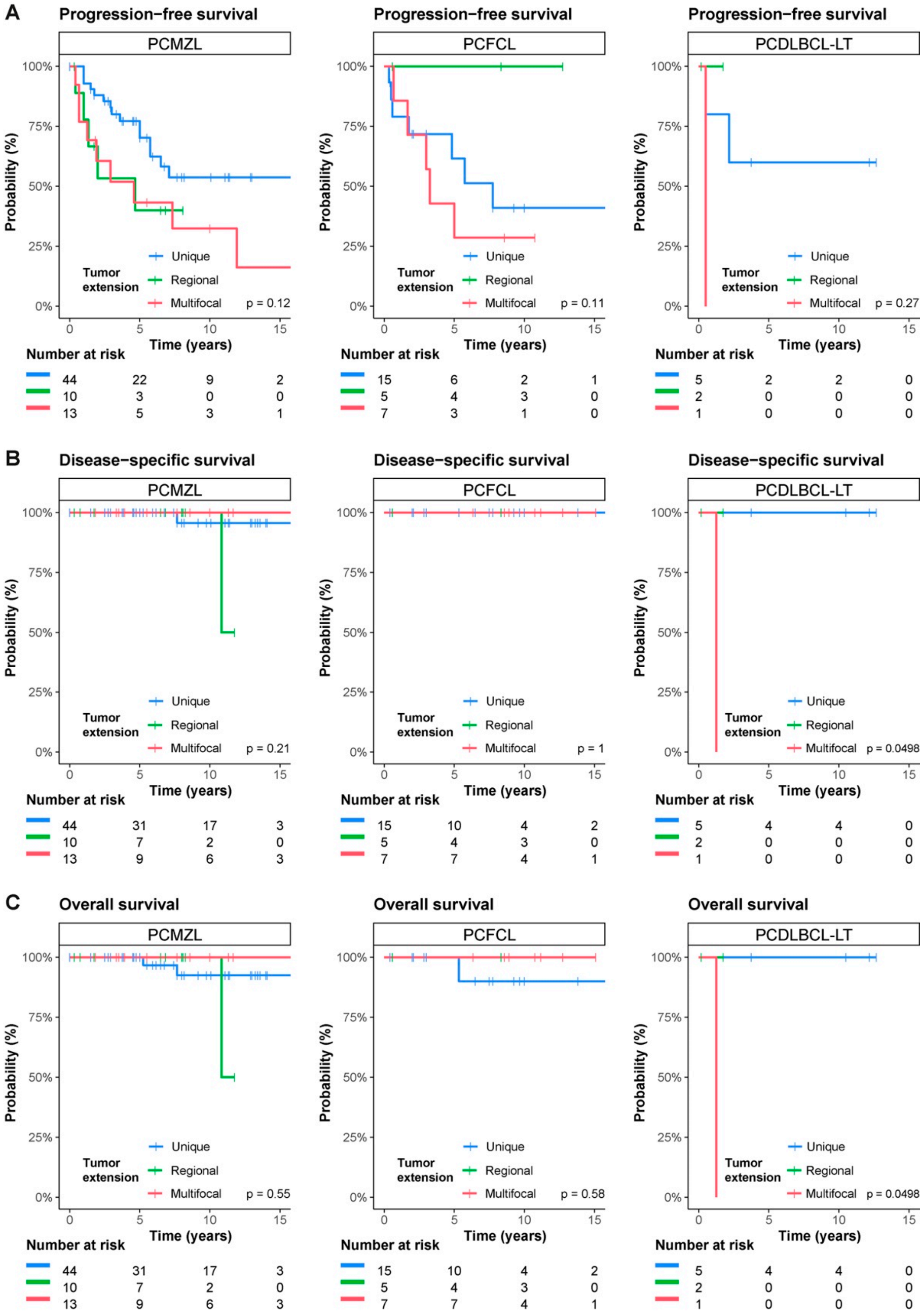

Figure S4: K–M survival curves according to the treatments received

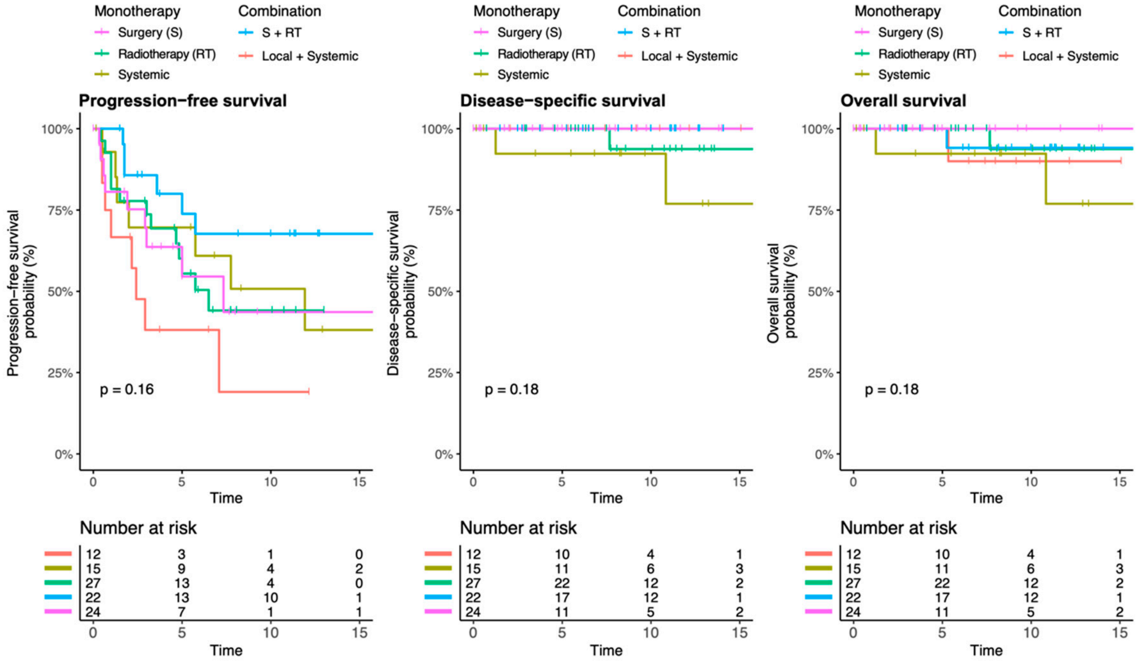

Figure S5: K–M survival curves according to local treatment only

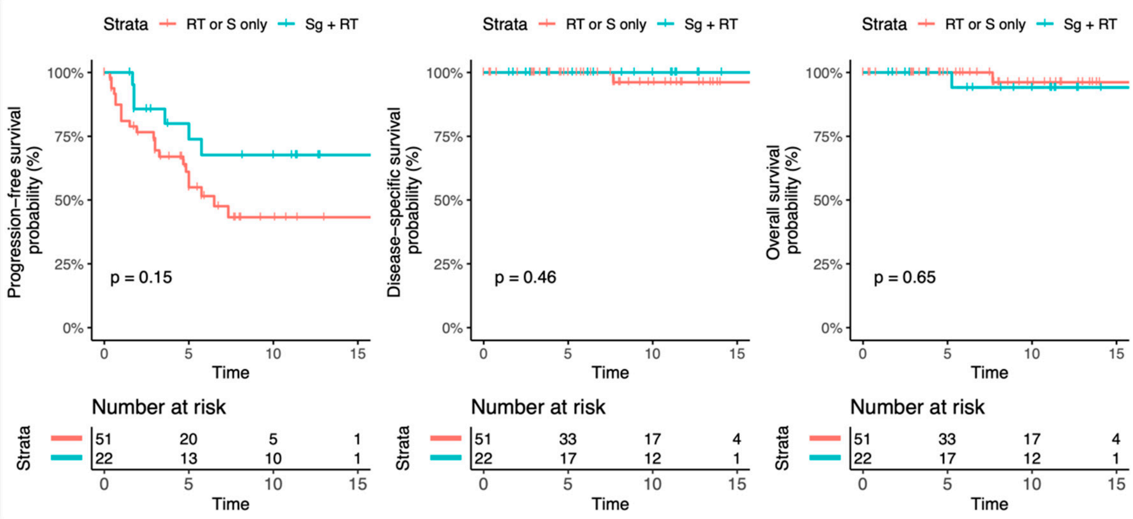

Supplement: Supplementary file 1 [file cancers-16-01034-s001.zip › cancers-2893059-supplementary.pdf]
